# The relationship of dental caries and weight status with adherence to school nutrition policies among public primary school children in Riyadh: a cross-sectional study

**DOI:** 10.1186/s12889-025-24979-0

**Published:** 2025-11-10

**Authors:** Areej Alsiwat, George Kitsaras, Anne-Marie Glenny, Haya Alayadi, Michaela Goodwin

**Affiliations:** 1 Division of Dentistry, School of Medical Science, Faculty of Biology, Medicine and Health, University of Manchester, Manchester, M13 9PL UK; 2 Dental Health Department, College of Applied Medical Sciences, King Saud University, PO Box10219, Riyadh, 11433 Saudi Arabia

**Keywords:** School canteen, Policy, Dental caries, Obesity

## Abstract

**Background:**

Since 2014, the Saudi Ministry of Health and Education has developed and required the implementation of the “Regulations of Health Conditions for School Canteens.” The Saudi Ministry of Education states these school food guidelines provide educational institutions a list of foods that are permitted as well as those that are prohibited, which they must adhere to. The aim of this research is to explore the relationship of dental caries and weight status with adherence to school food policy in public primary schools in Riyadh, Kingdom of Saudi Arabia (KSA).

**Methods:**

A cross-sectional study was conducted among public primary school children in Riyadh, KSA. A total of 14 schools were randomly selected from five different regions and identified in a previous study that explored school adherence to the school canteen policy set by the Ministry of Education in Riyadh, KSA. Seven schools that had an average adherence to the school canteen policy and seven schools that did not adhere were included in this study. The severity and prevalence of dental caries, as well as the prevalence of overweight and obesity in children, were assessed according to World Health Organization (WHO) criteria. Additionally, parents were asked to complete a structured questionnaire to evaluate their child’s oral health by using the WHO, Oral Health Questionnaire for Children.

**Results:**

This study found there is no evidence of an effect of adherence to school food policies on either the severity or prevalence of dental caries. Similarly, no evidence of an effect of adherence to school food policies on the prevalence of overweight and obesity among public primary schools in Riyadh, Saudi Arabia, with (IRR = 0.96, 95% CI: 0.80, 1.17, p-value = 0.714), (OR = 1.01, 95% CI: 0.605, 1.69, p-value = 0.960 ), and ( OR = 0.80, 95% CI: -0.57, 1.13, p-value = 0.207), respectively. However, based on parents’ perception of their children’s eating habits, there was evidence that children who ate biscuits and cakes at least once a week showed 78.2% lower odds of being caries free compared to once a month or never (OR = 0.21, 95% CI: 0.04, 1.00, *p* = 0.050). Additionally, children who consumed sugar-filled beverages daily or at least once a weekly had lower odds of being non-overweight/obese compared to those who consumed them once a month or never (OR = 0.63, 95% CI: 0.40, 0.97, *p* = 0.037) (OR = 0.63, 95% CI: 0.40, 0.98, *p* = 0.042).

**Conclusions:**

There was no evidence of a difference between the level of adherence to Ministry of Education school canteen guidelines in public primary schools and dental caries experience or weight status of students. However, due to the level of school canteen compliance being either ‘poor’ or ‘average’, a clear conclusion cannot be reached.

**Supplementary Information:**

The online version contains supplementary material available at 10.1186/s12889-025-24979-0.

## Background

Eating habits, specifically the intake of free sugars, cause many non-communicable diseases (NCDs) such as obesity, diabetes, heart disease, and tooth decay [1]. Dental caries, a prevalent dental disease, is mostly caused by poor dietary habits, especially excessive intake of sugar-containing foods [2]. Sugar is a major contributor to dental caries, but other factors, such as how often and when sugar is introduced, can make its impact on oral health much more noticeable [2]. In 2024, a meta-analysis was conducted to determine Saudi permanent and primary dental caries prevalence. Children aged 2–18 had their primary and permanent teeth assessed. They concluded that primary teeth had an average caries prevalence of 75.43% and a mean decayed-missing-filled teeth (dmft) score of 4.14 (95% CI: 3.11 to 5.18). Permanent teeth prevalence averaged 67.7%, with a mean of 1.28 (95% CI: 0.93 to 1.64) [3]. The study concluded that the current methods of prevention, such as water fluoridation, seem to be effective since the average dmft/DMFT scores for permanent and primary teeth were lower than in previous studies. However, one of the limitations of this study is that the included studies failed to control confounding factors, such as sociodemographic factors and parental education, which can influence children’s dental caries prevalence. The study also recommended that future research should focus on current school programs and ongoing education. The study also suggested continuing these interventions to maintain Saudi Arabia’s declining trend in caries prevalence [3].

Obesity and being overweight can be defined by an excessive or abnormal accumulation of fat that raises a person’s body weight over the recommended range and has a negative impact on their health. This increase results from an imbalance between the body’s need for energy and the energy obtained from eating [4]. In 2024, a previous systematic study looked at how common being overweight and obese is in Saudi Arabian children. The study included 21 Saudi Arabian research articles published between 2006 and 2023, which involved participants aged 2 to 19 years old. The research found that between 5% and 29% of people were overweight and between 3.8% and 49.7% were obese [5]. Furthermore, in a previous study conducted in Saudi Arabia, 10,735 adults (15 and above) took part in a multi-stage survey to identify factors that increase the likelihood of obesity. The data was collected using computer-assisted in-person interviews, with a focus on demographics, nutrition, physical activity, chronic disease, and health-related habits. The findings indicated an association between obesity and chronic noncommunicable diseases. This study indicated that obesity was associated with an increased risk of diabetes, hypertension, and high cholesterol [6]. To assess the efficacy of different school-based intervention programs and their influence on obesity, a systematic review was conducted. Three different databases (2010–2019) were screened to find primary and secondary school-based intervention programs that measured at least one of the following variables: physical activity, physical fitness, or obesity. The results of the study indicate that school-based intervention programs are an essential tool for promoting physical activity and lowering obesity [7].

The “Regulations of Health Conditions for School Canteens” were created by the Saudi Ministry of Health and Education in 2014 and have been in practice since then. According to the Saudi Ministry of Education, Saudi Arabia’s school food guidelines give school canteens a list of approved and forbidden items that they must follow [8]. Even though these guidelines have been mandated since 2014, there is no evidence that school canteen programs are playing a key role in reducing dental caries or the prevalence of overweight, and obesity among school-age children. The aim of this research is to explore the relationship of dental caries and weight status with adherence to school food policy in public primary schools in Riyadh, Kingdom of Saudi Arabia (KSA). The objectives are (a) assess the prevalence and severity of dental caries among public primary school children, (b) assess the prevalence of overweight and obesity among public primary school children, (c) explore the relationship between school food policy and the severity and prevalence of dental caries, as well as the prevalence of overweight and obesity (d) examine the relationship between a child’s eating habits with dental caries experience, and the prevalence of overweight and obesity (e) examine the relationship between a child’s brushing frequency and dental caries experience.

## Methodology

### Study design, setting, and participants

This study is a cross-sectional study set within public primary schools in Riyadh, Kingdom of Saudi Arabia.

### Inclusion criteria

• Children in 1 st, 2nd, or 3rd grade (6 to 9 years) and their parents.

## Exclusion criteria

• Children with chronic health conditions.
• Children under long-term medication, such as medication known to reduce salivary flow or sugar-containing medications.

### Training and calibration

Recruitment of dental hygienists was conducted through announcements on social media. Prior to the clinical examination, a total of 10 examiners and 10 recorders of dental hygienists received training and calibration. Each examiner was paired with a recorder, and the pair worked together throughout the study to ensure accuracy. They were trained in dental caries and BMI examinations. For dental caries, a two-day workshop was held and included theoretical and practical sessions. On day one, the examiners received theoretical training, which covered the WHO diagnostic criteria, followed by an exercise with visual slides of each clinical situation that could be encountered during the examination. Day two included a practical session involving 20 students aged 6 to 9 years. The examiner independently examined each participant under the same conditions. The examination took place in a clinical room, a designed space within schools used to provide health-related services to students, at one selected public primary school. Children were seated on chairs, and headlamps, disposable mouth mirrors, and tongue depressors were used for dental assessment. Calibration was performed using only the decayed component of the DMFT/dmft index, as untreated dental caries is the main public health problem and most prevalent in children in our population. Inter-rater reliability between examiners and the gold standard was assessed using the interclass correlation coefficient with a two-way effect model for absolute agreement. The average ICC measure among all examiners and the gold standard was > 0.9, indicating excellent agreement. During the practical session, the recorder also received training on height and weight measurements using a standardized measurement, as described in the data collection section.

### Sample size

A sample size calculation was carried out to ensure a sufficient sample could be achieved to determine the severity of dental caries and compliance with food policy guidelines (the primary outcome). The sample size calculation was performed, accounting for clustering within schools and an estimated difference of 1 DMFT (Decayed, Missing, and Filled Teeth) between groups (a mean of 4.4 DMFT in group 1 and 3.4 in group 2, with a sd. of 2.5). Assuming 60 children could be recruited from each school with an intra-class correlation coefficient (ICC —accounting for clustering) at 0.03 and a power of 90% would result in 7 schools and 420 per group (840 participants in total) [9]. A total of 14 schools were randomly selected from five different regions and included 7 schools that had average compliance with the school canteen policy and 7 schools that were poor in compliance. This data was based on a previous study that scored school canteens in public primary schools with a checklist set by the Ministry of Education. The alignment scores were determined by the Ministry of Education as either good, average, or poor based on their adherence to guidelines for the school canteen policy [10].

### Data collection

All parents of children eligible to participate in the schools received an information sheet and consent form along with a structured questionnaire in their children’s bags. The questionnaire focused on parents’ perceptions of their children’s oral health, using the WHO Oral Health Questionnaire for Children (2013) to gather this information [11]. The questionnaire was translated and then reviewed by an Arabic academic expert and piloted on 20 samples of parents not included in the study to verify its clarity; feedback was used to refine wording and ensure it was fully understood before data collection. After one week, a reminder email was sent to parents who had not returned their consent form and questionnaire (if lost, an extra copy was provided), and an acknowledgment was sent to those who agreed to participate. Parents were free to decline participation without explanation and were under no obligation to do so.

Clinical examination for dental caries severity and prevalence was performed according to the World Health Organization (WHO) criteria, which define dental caries at the caries into dentine threshold [11]. No radiographs were taken. Examinations were performed in empty clinical rooms; each child was examined one at a time, and only the examiner and recorder were present during the examination. Children were seated on a chair, and headlamps, disposable mouth mirrors, and tongue depressors were used for dental assessment. Standard infection control measures were in place.

The WHO chart was used to analyze the body mass index (BMI) of children. The WHO’s percentile cutoffs—obesity (beyond the 97th percentile), overweight (85th to 97th percentile), normal weight (15th to 85th percentile), thinness (3rd to 15th percentile), and extreme thinness (below the 3rd percentile)—were the basis for the categorization [12]. A calibrated platform electronic scale was used to measure the student’s weight while they were instructed to remove their heavy clothes and stand barefoot. Students were told to place their bodies evenly on both feet and stand in the center of the scale. The electronic scale is zeroed before each student is weighed, and it is calibrated by the researcher using a known weight after every 20th student. Height was measured using a stadiometer. Height measurement was then carefully read to the nearest 0.1 cm. The following formula was used to calculate BMI: Weight ^(kg)^/Height ^(cm)^. Children were not examined if they refused to be examined.

### Statistical analysis

SPSS version 29.0 was used for data analysis. Descriptive statistics (mean ± standard deviation and percentage) were used to summarize the social demographic factors, children’s oral health habits, the prevalence and severity of dental caries, and the prevalence of those within the category of overweight and obese according to their BMI. For children’s eating habits, the frequency of consumption has been categorized into three groups to simplify comparisons across various food items and to clarify the findings. “at least once or multiple times in a day” includes all responses that include consumption multiple times a day or daily; “at least once a week” includes responses that responded to consumption multiple times or once per week; and “at least once a month or never” includes responses that responded to monthly consumption or no consumption at all.

In addition, drinks with added sugar were grouped together as one single category (sugary drinks) to better highlight their overall impact and allow for clearer analysis of beverages containing added sugar. Furthermore, the Body Mass Index (BMI) for participants was divided into two primary groups: “healthy weight and thin” and “overweight and obese”. The “healthy weight and thin” group included those with BMIs in the normal weight, underweight, and extreme thin ranges; the “overweight and obese” group included participants with BMIs classified as overweight and obese in order to directly observe the possible risk factors for overweight and obesity. The generalized estimating equation (GEE) model with a negative binomial distribution was used to account for clustering and overdispersion (variance exceeds the mean) to examine factors associated with DMFT scores, such as dental caries experience and the association with adherence to the Ministry of Education policy and the child’s use of a toothbrush. The generalized estimating equations (GEE) with binomial distribution were performed to evaluate the association between dental caries, overweight and obesity (yes/no) with demographic factors (gender, age), socioeconomic (family income, parental education), and behavioral characteristics (sugar intake).

### Ethical approval

Data were encrypted and kept on servers or computers approved by the University of Manchester and backed up safely. All information related to this study was managed using unique ID numbers; during recruitment, ID numbers were issued, and they were used throughout this study. Anonymized data will be included in publications and conference presentations. All necessary ethical approvals were in place. This study obtained approval from the University Research Ethics Committee (UREC) (Ref. No. 2024–16282−32966), the Institutional Review Board at King Saud University (Ref. No. 23/0425/IRB), and the Ministry of Education in Saudi Arabia to both obtain a list of schools and access schools in Riyadh city (Ref. No. 4500028581).

## Results

### Demographic characteristics

Most of the characteristics of adhering and non-adhering schools are similar, based on demographic data (Table.1). The distribution of children between grades is almost the same, with around 33% of each grade level in each group. There is also a slight difference in participating parents’ gender, with females representing 54.5% in adhering schools and 59.3% in non-adhering schools. The average age of participating parents was 39.6 years in adhering schools and 38.8 years in non-adhering schools. Ethnicity is mainly Arab, with 100% in adhering schools and 99.3% in non-adhering schools. A total of 45.1% of parents have completed secondary school, 47.5% have a bachelor's degree, and postgraduate education is slightly more common in non-adhering schools (9% vs. 5.7%). Full-time employment stands at 49.8% in adhering schools and 50% in non-adhering schools, while unemployment is at 41.4% and 37.8%, respectively. Thus, employment status is nearly the same. Although there is a slightly greater percentage of families in the 10,000–15,000 income range in non-adhering schools (25.4% vs. 15.7%), the distribution of family incomes is generally similar. Despite that there are many similarities between adhering and non-adhering schools, there is a noticeable difference in the gender distribution of the students: students in adherent schools were 98.1% female and 1.9% male, compared to 81.4% female and 18.6% male at non-adhering schools.

**Table 1:** Demographic characteristics

		Adhering Schools	Non-Adhering Schools	Overall Schools
Child Gender	*Female*	98.1%	81.4%	89.8%
	*Male*	1.9%	18.6%	10.2%
Child Grade	*1* ^st^ * Grade*	33.1%	33.3%	33.2%
	*2* ^nd^ * Grade*	32.9%	33.3%	33.1%
	*3* ^rd^ * Grade*	33.8%	33.3%	33.7%
Parent Gender	*Female*	54.5%	59.3%	57.1%
	*Male*	45.0%	40.7%	42.9%
Parent Age	*Mean* *SD*	39.62 7.41	38.77 7.32	39.3 6.34
Relationship with child	*Mother *	55.2%	56.7%	56.1%
	*Father *	42.1%	39.8%	41.0%
	*Grandparent *	0.5%	1.2%	0.8%
	*Guardian *	1.9%	2.4%	2.1%
Ethnicity	*Arab*	100%	99.3%	99.6%
	*Asian*	-	0.7%	0.4%
Educational Level	*≤Secondary school*	43.1%	44.8%	45.1%
	*Bachelor*	49.8	45.2%	47.5%
	*Postgraduate*	5.7%	9%	7.4%
Employment Status	*Full time employed*	49.8%	50%	49.9%
	*Self employed*	2.1%	5.5%	3.5%
	*Part time employed*	5.7%	5%	5.4%
	*Student*	0.2%	1.4%	0.8%
	*Unemployed*	41.4%	37.8%	40.5%
Total Family Income	*Less than 2500 *	10%	7.9%	8.9%
	*2500 ≥ 5000 *	25.7%	22.8%	24.3%
	*5000 ≥ 10000 *	30.5%	27.8%	31.3%
	*10000 ≥ 15000 *	15.7%	25.4%	20.6%
	*Above 15000 *	16%	13.8%	14.9%

### The relationship of dental caries, overweight, and obesity with adherence to the school nutrition policies

The prevalence of dental caries among children was 79.6%. (79.5% for average adhering schools and 79.8% for schools not adhering). In terms of severity, the mean decayed-missing-filled teeth (DMFT) index was 4.38 (95% confidence interval: 4.15–4.61), with 4.42 for average adhering schools (95% confidence interval: 4.09–4.75) and 4.34 for non-adhering schools (95% confidence interval: 4.01–4.66). The overweight prevalence was 13.7%, and obesity prevalence was 7.1%, with a combined prevalence of 20.8% (19.3% average adherence, 22.4% non-adherence). After adjustment of parent sociodemographic factors and child eating habits in all models and brushing frequency in the dental caries models, the results for this study found no evidence of a difference between alignment with school food policies and the dependent variable (DMFT) (IRR = 0.96, 95% CI: 0.80, 1.17, p-value = 0.714), the occurrence of dental caries (yes/no) (OR = 1.01, 95% CI: 0.60, 1.69, p-value = 0.960), or the prevalence of overweight and obesity (OR= 0.80, 95% CI: −0.57, 1.13, p-value = 0.207) (Table.2).

**Table 2:** Relationship of dental caries, overweight, and obesity with adherence to the school nutrition policies

**Outcome**	***IRR***	**95% CI ** **(Lower, Upper)**	**p-value**
DMFT	0.96	(0.80, 1.17)	0.714
Dental Caries (yes/no)	***OR ***1.01	(0.60, 1.69)	0.960
Overweight and obesity (yes/no)	***OR ***0.80	(−0.56, 1.13)	0.207

However, important sociodemographic variables showed evidence of a relationship with a child's dental caries and weight status. For dental caries severity, the study found strong evidence that children in grade 1 had an incidence rate of DMFT that was 21.5% lower compared to children in grade 3 (IRR= 0.78, 95% CI: 0.66, 0.93, p=0.004). In addition, other variables such as the number of children in the household and family income showed children with higher incident rates of DMFT. With every increase in one child in the household, the incidence rate of DMFT increases by 5.1% (IRR=1.05, 95% CI: 1.01, 1.09, p=0.018). In addition, total family income has shown that children with family incomes lower than 15000 Riyal had higher incident rates of DMFT (IRR= 1.94, CI: 1.48, 2.54, p < 0.001), (IRR = 1.52, CI: 1.31, 1.75, p < 0.001), (IRR = 1.52, CI:1.29, 1.78, p < 0.001), and (IRR = 1.34, CI: 1.15, 1.56, p < 0.001) (Additional file 1).

Furthermore, the relationship between demographic characteristics and the occurrence of dental caries (yes/no) showed evidence of a difference between children in grade 1 and grade 3, with children in grade 1 having 64.7% higher odds of being caries free compared to children in grade 3 (OR = 1.64, 95% CI: 1.10, 2.46, p = 0.015). Children whose parents worked full-time had 35.5% lower odds of being caries free compared to those whose parents were unemployed, homemakers, or retired (OR = 0.65, 95% CI: 0.43, 0.96, p = 0.032). Family income was strongly related to caries: the evidence suggested children from families earning less than 15000 Riyal had lower odds of being caries free compared to families earning above 15000 Riyal. The strongest association was found in income below 2500 Riyal (OR = 0.12, 95% CI: 0.04, 0.37, p < 0.001), followed by families earning 2500–5000 Riyal (OR = 0.35, 95% CI: 0.23, 0.54, p < 0.001), 5000–10000 Riyal (OR = 0.25, 95% CI: 0.14, 0.43, p< 0.001), and 10000–15000 Riyal (OR = 0.41, 95% CI: 0.23, 0.70, p = 0.001). Finally, for each additional child under the age of 16 in a household, the odds of a child being caries free lower by 23.2% (OR= 0.76, 95% CI: 0.65, 0.89, p<0.001) (Additional file 2).

For the prevalence of overweight and obesity, part-time employed parents had higher odds of children non-overweight/obese compared to employed parents (OR =4.95, 95% CI: 1.11, 21.88, p=0.035). Families making less than 15,000 Riyal showed higher odds of children non-overweight/obese compared to families making over 15,000 Riyal, which might suggest that overweight and obesity are more common among those with higher incomes (OR=1.57, 95% CI: 1.08, 2.28, p=0.017). Additionally, for each additional child under the age of 16 in a household, the odds of non-overweight/obese children lower by 14.1% (OR= 0.85, 95% CI: 0.74, 0.99, p=0.042) (Additional file 3).

### Child dental health and eating habits

To explore children's dental health and eating habits, a total number of 840 parents agreed to fill out the questionnaire. When asked how often their children brush their teeth, 21.0% of parents said they do so twice a day, 47.3% said they do so once a day, and 31.7% said they brush less than once a day. A toothbrush was used by 98.2% of the children, and only 6.1% use dental floss. A percentage of 97.7% of the children use toothpaste, 57.3% of the children's toothpaste contains fluoride, and 25.6% don’t know. This study found no evidence of a relationship between brushing frequency and the prevalence or severity of dental caries with p-values >0.05 (Table.3). This could be a result of social desirability bias, as participants may overreport for social acceptance.

**Table 3:** The relationship between brushing frequency with the prevalence or severity of dental caries

			**DMFT**			**Dental caries yes/no**	
	**Reference**	**IRR**	**95% CI ** **(** **Lower, Upper** **)**	**p-value**	**OR**	**95% CI ** **(** **Lower, Upper** **)**	**p-value**
Several times a day	less than once a day	1.05	(0.92, 1.19)	.4570	0.81	(0.54, 1.21)	0.315
Once a day		0.96	(0.84, 1.10)	0.607	0.98	(0.61, 1.59)	0.956

In addition, parents were questioned about the frequency of their children's eating habits. The frequency of fresh fruit consumption among children was 25.1% for daily consumption and 42.4% for several times per week. With a proportion of 40.2%, the parents reported consuming cariogenic foods such as cakes and biscuits many times a week, while 33.6% reported consuming them daily. Additionally, 46.7% of parents said their children eat sweet candy many times a week, while 21.5% said they only consume it once a week. A total of 34.0% of respondents said they consume sugary beverages many times a week, and 22.5% said they consume them every day. Regarding the relationship between eating habits and dental caries, only eating biscuits at least once a week showed 78.2% lower odds of being caries free compared to once a month or never (OR = 0.21, 95% CI: 0.04, 1.00, p = 0.050).

Furthermore, compared to once a month or never, children consuming daily lemon juice/soft drinks or at least once a week honey/jam showed lower odds of children being non-overweight/obese (OR=0.47, 95% CI: 0.29, 0.77, p=0.003) (OR=0.62, 95% CI: 0.47, 0.83, p=0.001). Daily or at least once a week consumption of sugar-filled beverages resulted in children with lower odds of being non-overweight/obese compared to those consuming once a month or never (OR=0.63, 95% CI: 0.40, 0.97, p=0.037; OR=0.63, 95% CI: 0.40, 0.98, p=0.042). Contrary to expectation, children consuming biscuits or cakes daily or at least once a week had higher odds of being non-overweight/obese compared to never or once a month (OR = 2.07, 95% CI: 1.10, 3.88; p = 0.022) (OR = 3.05, 95% CI: 1.49, 6.22, p = 0.002).

## Discussion

The current study examined the relationship of dental caries and weight status with adherence to school food policies in Riyadh, Saudi Arabia. The study indicated that there was no evidence of a difference in dental caries, weight status between schools that had an average alignment with the school food policy established by the Ministry of Health and Education and those that did not. This finding contrasts with a previous study conducted in Brazil, which found that nursery dietary guidelines effectively implemented menus with lower sugar content, resulting in a lower likelihood of developing dental caries [13]. In addition, a study conducted in the USA concluded the schools with nutrition-focused wellness policies had significantly lower BMI increases in middle school students [14]. However, because schools in this study only had either an average or poor compliance with the school canteen policy, a clear conclusion cannot be drawn about its impact on children, highlighting the importance of effective school canteen implementation to ensure better results.

The prevalence and severity of dental caries were initially evaluated in this study. The mean decayed-missing-filled teeth (DMFT) index for dental caries severity was 4.38 (95% CI 4.15–4.61), while the prevalence of dental caries among children was 79.6%. This finding is comparable to a 2024 meta-analysis that reported an average dental caries prevalence of 75.43 percent across the Kingdom of Saudi Arabia, along with a mean decayed-missing-filled teeth score of 4.14 [3]. These similarities suggest that dental caries has remained an issue in children, and increased focus on preventative and educational initiatives is required to enhance dental health. This study also evaluated the prevalence of obesity and overweight children, finding that the rates were 7.1% and 13.7%, respectively. These results are consistent with a previous retrospective study on 351,195 children and adolescents in Saudi Arabia between 2016 and 2021 who were between the ages of 2 and 19, which found that almost 20% of the population was either overweight (11.2%) or obese (9.4%) [15]. Additionally, Saudi Arabian studies published between 2006 and 2023 with 63,512 participants ages 2 to 19 were included in a systematic review carried out in 2024. The study found that the prevalence of obesity ranged from 3.8% to 49.7%, whereas the prevalence of overweight ranged from 5% to 29% [5]. These findings suggest that overweight and obesity rates among Saudi Arabian primary school students remain quite high, highlighting the continued negative effects of poor eating habits and sedentary lifestyles as well as the need for more focused, efficient public health initiatives. However, due to the nature of public primary schools in Riyadh, Saudi Arabia, where 1 st to 3rd grades can be mixed gender in female schools or boys only in male schools, depending on parents' preference, there was a gender imbalance. The overall prevalence of dental caries and obesity may have been influenced by gender distribution, as there was a higher proportion of female than male students. Previous research has shown that dental caries and obesity prevalence may differ in genders due to behavior and biological factors [16–18], which must be considered when interpreting the results of this study.

This study additionally examined the relationship between the prevalence of dental caries and socioeconomical factors. The results indicated that factors such as the child's grade level, the number of children living in the household, employment status, and the total family income showed evidence of an associated with the occurrence of dental cavities. These findings are consistent with prior retrospective research that examined first- and fourth-grade students from public and private schools in Riyadh, Saudi Arabia. The study discovered that fourth-grade students were more likely than first-grade students to have dental caries [19]. These findings align with expectations, as dental caries is a progressive disease, and once dentinal decay develops, it doesn’t reverse. Furthermore, according to the same study, caries was also more common in girls than in boys [19]. However, the current study did not identify a significant difference in caries prevalence across genders due to the varying sample sizes of boys and girls, with girls making up 89.8% and boys 10.2% of the participants. This study identified the number of children in a family as a factor associated with the prevalence of dental caries, consistent with previous research indicating that larger families may face greater challenges in accessing dental care [20]. In addition, fully employed parents are more likely to have children with caries compared to unemployed parents; this contrasts with a previous study carried out on preschool children in Riyadh, Saudi Arabia. The study found that full-time parents are less likely to have children with dental caries [21]; however, this finding may be influenced by the fact that most households had two parents but the parent who completed the questionnaire may have been unemployed. Furthermore, household income was an important factor, as families with lower incomes had a higher prevalence of caries. This finding supports previous research indicating a link between low household income and a higher prevalence of dental caries [22].

For caries severity, child grade, number of children in household, and family income were found to be significant factors. This was consistent with a previous study that aimed to estimate the prevalence and severity of dental caries among schoolchildren aged 9 to 12 in Al-Madinah, Saudi Arabia. A cross-sectional survey on oral health and dental caries assessment was conducted in both public and private primary schools, utilizing the DMFT/dmft index for assessment. The findings revealed that older children in the study group had higher average DMFT score than their younger students. Additionally, males, Saudi nationals, children attending public schools and those from low-income households had higher caries severity [23]. Furthermore, a simple random sampling method was used to select 300 children between the ages of 6 and 12 for descriptive analytical study [24]. The association between dmft/DMFT scores and the impact of influencing variables on dental caries was assessed. According to the study, DMFT scores increased as the number of children in the family increased, consistent with the findings of this study. However, the study also found that DMFT scores decreased with more frequent brushing [24], which was inconsistent with the findings of this study, as this study found no evidence of a relationship between DMFT scores and the frequency of brushing. This discrepancy could be due to the participants' sugary diet, which leads to high rates of DMFT and caries occurrence. Other factors, such as parents overestimating their children's frequency of brushing for social desirability or the technique used for brushing and if fluoridated toothpaste is used, are also important factors to understand the impact of brushing on the DMFT scores.

Furthermore, this study also evaluated the relationship between demographic characteristics and weight status, revealing a positive association with the number of children living in the household. Similarly, in Jeddah, Saudi Arabia, children and adolescents aged two to eighteen participated in a cross-sectional study. Medical experts included and questioned a total of 521 children. The results indicated that a higher body mass index (BMI) is associated with households including more than four members in the family [25]. The chance of having a higher BMI with a bigger family size could be due to less time parents have to monitor each child's diet. Additionally, this study showed that unemployed parents are more likely to have overweight or obese children compared to part-time employed parents. This contrasted with a previous study that involved 240 male students between the ages of 7 and 15 using stratified random sampling in Al-Ahsa, Saudi Arabia. Determining the prevalence of obesity and its risk factors was the aim of the study. The study found that employed mothers had a higher prevalence of obese children [26]. However, the fact that most households had two parents while the parent who answered the questionnaire might not have been employed may have influenced this result.

This study also found those with a higher family income are more likely to have obese children. Similarly, 384 obese children aged 3 to 18 participated in previous cross-sectional research that was carried out in Jeddah. The study found that parents' education and household income were associated with their children's obesity prevalence; a higher prevalence of obesity was shown to be associated with parents with higher levels of education and household income. The results also demonstrated differences between genders, with male individuals showing a higher prevalence of obesity than female [27]. This couldn't be determined in the current study, as there was a significant difference in the sample size across genders.

Lastly, parents were questioned about their children's eating habits. According to the findings, children consumed the following foods and beverages most frequently throughout the week: fresh fruits (42.4%), cakes and biscuits (40.2%), sweet candy (46.7%), and sugary drinks (34.0%). The results of this survey are in line with a 2018 study carried out in Saudi Arabian primary schools for boys, which found that children consumed the following foods at least twice a week: 29.4% consumed fresh fruit, 83.4% consumed sweets, 89.9% drank flavored juices, 35.6% drank flavored milk, and 83.4% consumed chips [22]. This highlights an alarming pattern in children's eating patterns, especially regarding food and drink that may lead to dental problems. Furthermore, according to this study, consuming drinks with added sugar and soft drinks like Coca-Cola or lemon weas significantly associated with being overweight or obese. This finding is consistent with a study conducted in Riyadh, Saudi Arabia, which found that sugar-sweetened carbonated drinks (SCB) were associated with higher BMI in both male and female students between the ages of 10 and 19 years [28]. The association between consuming sugary snacks and beverages and being overweight or obese may be explained by people choosing these quick and appealing meals over healthier alternatives. Additionally, this can result in insufficient consumption of nutritious meals, raising an individual's body mass index. This highlights the need for better nutritional education to help children, and their parents make better dietary choices, especially by increasing fresh fruit consumption and lowering cariogenic intake for better dental and overall health. Furthermore, due to children's poor nutritional practices based on this study and previous research, schools must pay attention to children's homemade meals.

### Strengths

This study is the first to compare dental caries, weight status between schools that adhere with the Ministry of Education's school food policy and those that do not, and it includes a broad variety of schools from different regions. Furthermore, another study's strength is its large sample size along with its high retention rate.

### Limitations

Since school canteen policy compliance was either average or poor, it is hard to tell how this may affect students' dental and general health. The study's cross-sectional design limits the ability to draw conclusions about cause and effect. A longitudinal study is required to establish a causal association, even if this research did not discover any relationship. Another limitation of this study is the obvious gender imbalance in our sample, since 89.8% of the participants are female. Our findings may not be generalizable to the general population, particularly boys, due to this imbalance. The results may be more indicative of this group due to the higher number of female participants, even though we controlled for a variety of socioeconomic variables in our investigations. Research in the future must aim for a more representative sample of the population at large if we are to get any insight into potential gender-based effects. The study's limitations include its exclusive focus on Riyadh's public primary schools, making its findings inapplicable to other Saudi Arabian cities' public elementary, or middle, or private schools.

### Recommendation

Taking into account the length of time that schools have been following the canteen guidelines, longitudinal studies may provide useful information about the impact of schools' compliance with the guidelines on the changes in dental caries and body mass index (BMI). The impact of changes at home and in the school environment on children's dental and overall health should be carefully considered. As a whole, home-brought meals, snacking habits, and regular dental practices all contribute. An approach that involves the entire community and family might lead to better dental and overall wellness outcomes.

## Conclusion

There was no evidence of a difference between the level of adherence to Ministry of Education school canteen guidelines in public primary schools and dental caries experience or weight status of students. However, due to the level of school canteen compliance being either ‘poor’ or ‘average’, a clear conclusion cannot be reached. It is suggested that the effective implementation of school canteen guidelines needs to be supported, and educational programs for parents to promote and improve dental and nutritional behaviors in families need to be provided with precise dietary guidelines for homemade meals.

## Supplementary Information

Supplementary Material 1.

Supplementary Material 2.

Supplementary Material 3.

## Data Availability

The datasets used and/or analyzed during the current study are available from the corresponding author on reasonable request.

## Abbreviations

KSA: Kingdome of Saudi Arabia
WHO: World Health Organization
NCDs: Non-communicable diseases
OHRQoL: Oral Health-Related Quality of Life
BMI: Body mass index
DMFT: Decayed, Missing, and Filled Teeth
SCB: Sugar-sweetened carbonated drinks

## References

[1] World Health Organization. Guideline: sugars intake for adults and children. Geneva: World Health Organization; 2015. 25905159

[2] Feldens CA, Kramer PF, Vargas-Ferreira F. The role of diet and oral hygiene in dental caries. In: Fragelli CMB, editor. Pediatric restorative dentistry. Cham: Springer International Publishing; 2018. p. 31–55.

[3] Qadir Khan S, Alzayer HA, Alameer ST, Ajmal Khan M, Khan N, AlQuorain H, et al. Sequel: prevalence of dental caries in Saudi Arabia: a systematic review and meta-analysis. Saudi Dent J. 2024;36:963–9. 39035563 10.1016/j.sdentj.2024.04.011 PMC11255963

[4] Ministry of Health Saudi Arabia. *Obesity Awareness Platform* [Internet]. Riyadh: Ministry of Health; [cited 2025 Jan 5]. Available from: https://www.moh.gov.sa/en/AwarenessPlatform/ChronicDisease/Pages/Obesity.aspx

[5] Adam TR, Hamed AM, Saad M, Mohammed H, Elryahi Elsayed Elshareef T, Mushaeb H, et al. Prevalence of childhood obesity among children and adolescents in Saudi arabia: a systematic review. Cureus. 2024;16(9):e70135. 10.7759/cureus.70135. 39463548 10.7759/cureus.70135 PMC11502987

[6] Memish ZA, El Bcheraoui CE, Tuffaha M, Robinson M, Daoud F, Jaber S, et al. Obesity and associated factors - Kingdom of Saudi Arabia, 2013. Prev Chronic Dis. 2014;11:E174. 25299980 10.5888/pcd11.140236 PMC4193060

[7] Yuksel HS, Şahin FN, Maksimovic N, Drid P, Bianco A. School-based intervention programs for preventing obesity and promoting physical activity and fitness: a systematic review. Int J Environ Res Public Health. 2020;17(1):347. 10.3390/ijerph17010347. 31947891 10.3390/ijerph17010347 PMC6981629

[8] Aldubayan K, Murimi M. Compliance with school nutrition policy in Saudi arabia: a quantitative study. East Mediterr Health J. 2019;25(4):230–8. 31210343 10.26719/emhj.18.034

[9] Alayadi H, Sabbah W, Bernabé E. Effectiveness of school dental screening on dental visits and untreated caries among primary schoolchildren: study protocol for a cluster randomised controlled trial. Trials. 2018;19:214. 29653545 10.1186/s13063-018-2619-2 PMC5899334

[10] Alsiwat A, Kitsaras G, Glenny AM, Alayadi H, Goodwin M. Public primary school compliance with school canteen policy in Riyadh, Saudi Arabia: a cross-sectional study. Nutrients [Internet]. 2025;17(5):854. Available from: https://www.mdpi.com/2072-6643/17/5/854 10.3390/nu17050854 PMC11901446 40077725

[11] World Health Organization. Oral health surveys: basic methods. 5th ed. Geneva: World Health Organization; 2013.

[12] World Health Organization. (2020): Body mass index-for-age (BMI-for-age), [Internet]. Geneva: World Health Organization; 2020 [cited 2021 Sep 24]. Available from: https://www.who.int/tools/growth-reference-data-for-5to19-years/indicator-development

[13] Rodrigues CS, Watt RG, Sheiham A. Effects of dietary guidelines on sugar intake and dental caries in 3-year-olds attending nurseries in Brazil. Health Promot Int. 1999;14(4):329–35.

[14] Ickovics JR, Duffany KOC, Shebl FM, Peters SM, Read MA, Gilstad-Hayden KR, et al. Implementing school-based policies to prevent obesity: cluster randomized trial. Am J Prev Med. 2019;56(1):e1–11. 30573151 10.1016/j.amepre.2018.08.026 PMC7050629

[15] Alenazi S, Alajlan R, Alkhalaf H, Abolfotouh M, Alharbi O, Alfawaz R, et al. Prevalence of obesity among children and adolescents in Saudi arabia: a multicenter population-based study. Saudi J Med Med Sci. 2023;11(1):19–25. 36909009 10.4103/sjmms.sjmms_417_22 PMC9997853

[16] Mohamed RN, Hussein YM, Shamrani A, Bassuoni AS, Mohamed MW, Mohamed RN et al. Caries Prevalence and Treatment Need Among Primary School Children in Taif, Saudi Arabia [Internet]. Article in Indian Journal Of Applied Research. 2015; 5 (7). Available from: https://www.researchgate.net/publication/284168038

[17] Madhuri ASN, Kalla K. Discrepancy in caries prevalence by gender among school-going children in the population of Hyderabad: a cross-sectional study. Int J Sci Healthc Res. 2023;8(4):344–9.

[18] Al-Hazzaa HM, Alrasheedi AA, Alsulaimani RA, Jabri L, Alhowikan AM, Alhussain MH, et al. Prevalence of overweight and obesity among Saudi children: a comparison of two widely used international standards and the National growth references. Front Endocrinol (Lausanne). 2022;13:954755. 36004353 10.3389/fendo.2022.954755 PMC9393362

[19] Almutairi B, Adam TR, Bustami R. Caries prevalence among children at public and private primary schools in Riyadh: a retrospective study. BMC Oral Health. 2024;24:809. 39020334 10.1186/s12903-024-04570-6 PMC11256497

[20] Sabbagh HJ, Aljehani SA, Abdulaziz BM, Alshehri NZ, Bajkhaif MO, Alrosini SK, et al. Oral health needs and barriers among children in Saudi Arabia. Int J Environ Res Public Health. 2022. 10.3390/ijerph192013584. 36294162 10.3390/ijerph192013584 PMC9603417

[21] Al-Meedani LA, Al-Dlaigan YH. Prevalence of dental caries and associated social risk factors among preschool children in Riyadh, Saudi Arabia. Pak J Med Sci. 2016;32(2):452–6. 27182260 10.12669/pjms.322.9439 PMC4859043

[22] Alhabdan YA, Albeshr AG, Yenugadhati N, Jradi H. Prevalence of dental caries and associated factors among primary school children: a population-based cross-sectional study in Riyadh, Saudi Arabia. Environ Health Prev Med. 2018;23(1):60. 10.1186/s12199-018-0750-z. 30497366 10.1186/s12199-018-0750-z PMC6267843

[23] Aqeeli A, Alsharif AT, Kruger E, Tennant M, Bakeer H. Caries prevalence and severity in association with sociodemographic characteristics of 9-to-12-year-old school children in Al-Madinah, Saudi Arabia. Saudi Dent J. 2021;33(8):897–903. 34938031 10.1016/j.sdentj.2021.09.008 PMC8665159

[24] Soleimani M, Amini N, Askarizadeh N. Association of body mass index and DMFT/dmft in children aged 6–12 years. J Res Dent Maxillofac Sci. 2023;8(2):95–101.

[25] Al-Agha AE, Tatwany BO, Aiash DA. The effect of socioeconomic status, number of siblings and parental education on children body mass index at Jeddah, Saudi arabia: cross sectional study. Fam Med Med Sci Res. 2015;04(05).

[26] Albin Saleh AA, Alhaiz AS, Khan AR, Al-Quwaidhi AJ, Aljasim M, Almubarak A, et al. Prevalence of obesity in school children and its relation to lifestyle behaviors in Al-Ahsa district of Saudi Arabia. Glob J Health Sci. 2017;9(12):80.

[27] Mesawa A, Almutairi A, Abdullah A, Kutbi R, Almarri A, Alahdali H, et al. Parental socioeconomic status and occupation in relation to childhood obesity. Int J Med Developing Ctries. 2020;4(3):576–85.

[28] Collison KS, Zaidi MZ, Subhani SN, Al-Rubeaan K, Shoukri M, Al-Mohanna FA. Sugar-sweetened carbonated beverage consumption correlates with BMI, waist circumference, and poor dietary choices in school children. BMC Public Health. 2010;10:234. 10.1186/1471-2458-10-234. 20459689 10.1186/1471-2458-10-234 PMC2877673

