# Supplementary material for: The relationship of dental caries and weight status with adherence to school nutrition policies among public primary school children in Riyadh: a cross-sectional study
Source: BMC Public Health. 2025 Nov 10;25:3871. doi: 10.1186/s12889-025-24979-0 (PMC12604184; doi:10.1186/s12889-025-24979-0)
Supplement: Supplementary file 1 — Supplementary Material 1. [file 12889_2025_24979_MOESM1_ESM.docx]

Additional file 1**:** Negative Binomial Analysis of Factors Associated with DMFT (GEE)

| **Predictor** | **Category** | **Reference**  **Category** |  | **IRR** | **95% CI**  **(Lower, Upper)** | **p-value** |
| --- | --- | --- | --- | --- | --- | --- |
| Alignment | Not Aligned | Aligned |  | 0.96)) | (0.80, 1.17) | 0.714 |
| Child Grade | Grade 1 | Grade 3 |  | 0.78)) | (0.66, 0.93) | 0.004* |
|  | Grade 2 |  |  | 0.90)) | (0.80, 1.02) | 0.120 |
| Parent Gender | Female | Male |  | (1.36) | (1.091, 1.68) | *0.005 |
| Parent age | Continuous variable | - |  | (0.99) | 0.98, 1.01)) | .4390 |
| Relationship with child | Guardian | Mother |  | (1.24) | 0.87, 1.76)) | .2160 |
|  | Grandparent |  |  | (0.86) | 0.33, 2.20)) | .7560 |
|  | Father |  |  | (1.30) | 1.02, 1.64)) | .0280 |
| Education level | Postgraduate | ≤Secondary School |  | (1.045) | (0.74, 1.45) | .7950 |
|  | Bachelor |  |  | (0.91) | (0.79, 1.04) | .1950 |
| 1. Employment status | 1. Full-time employment | Unemployed / Homemaker / Retired |  | (1.13) | (0.98, 1.29) | 0.071 |
|  | 1. Self-employed |  |  | (1.23) | (0.96, 1.57) | 0.088 |
|  | 1. Part-time employment |  |  | (1.09) | (0.85, 1.40) | 0.454 |
|  | 1. Student |  |  | (1.09) | (0.92, 1.30) | 0.300 |
| Adults currently live in your household | One adult |  |  | (1.03) | (0.86, 1.24) | .7070 |
|  | Two adults | More than four adults |  | (1.02) | (0.86, 1.20) | .7990 |
|  | Three adults |  |  | (1.13) | (0.96, 1.35) | .1340 |
|  | Four adults |  |  | (1.07) | 0.92, 1.25)) | .3320 |
| Number of children <16 years | Continuous variable | - |  | (1.05) | (1.01, 1.09) | 0.018* |
| Total Family Income | Less than 2500 | Above 15,000 |  | (1.94) | (1.48, 2.54) | <.001* |
|  | 2500–5000 |  |  | (1.52) | (1.31, 1.75) | <.001* |
|  | 5000–10000 |  |  | (1.52) | (1.29, 1.78) | <.001* |
|  | 10000–15000 |  |  | (1.34) | 1.15, 1.56)) | <.001* |
| How often does your child brush their teeth |  | Less than once a day |  |  |  |  |
|  | Several times a day |  |  | (1.05) | (0.92, 1.19) | .4570 |
|  | Once a day |  |  | (0.96) | (0.84, 1.10) | 0.607 |
| Biscuits & Cakes consumption | At least once a day | Once a month or never |  | (1.47) | (0.91, 2.38) | .1120 |
|  | At least once a week |  |  | (1.52) | (0.93, 2.47) | 0.089 |
| Fresh fruit  consumption | At least once a day | Once a month or never |  | (1.29) | (0.84, 1.99) | .2380 |
|  | At least once a week |  |  | (1.44) | 0.93, 2.22)) | 0.101 |
| Jam/honey  consumption | At least once a day | Once a month or never |  | (0.94) | 0.77, 1.16)) | .6150 |
|  | At least once a week |  |  | (1.06) | 0.93, 1.20)) | .3440 |
| Sweets/candy  consumption | At least once a day | Once a month or never |  | (0.88) | (0.68, 1.13) | .3340 |
|  | At least once a week |  |  | (0.89) | (0.74, 1.08) | .2560 |
| Chewing gum containing sugar  consumption | At least once a day | Once a month or never |  | (1.05) | (0.84, 1.31) | .6490 |
|  | At least once a week |  |  | (0.99) | (0.89, 1.09) | .8910 |
| Lemonade, Coca Cola or other soft  drinks consumption | At least once a day | Once a month or never |  | (1.08) | (0.84, 1.38) | .5230 |
|  | At least once a week |  |  | (0.99) | (0.86, 1.14) | .9650 |
| Sugary drinks consumption | At least once a day | Once a month or never |  | (1.01) | (0.87, 1.15) | .9290 |
|  | At least once a week |  |  | (0.96) | (0.83, 1.12) | .6420 |

**^*p<0.05^**
