# Supplementary material for: The relationship of dental caries and weight status with adherence to school nutrition policies among public primary school children in Riyadh: a cross-sectional study
Source: BMC Public Health. 2025 Nov 10;25:3871. doi: 10.1186/s12889-025-24979-0 (PMC12604184; doi:10.1186/s12889-025-24979-0)
Supplement: Supplementary file 2 — Supplementary Material 2. [file 12889_2025_24979_MOESM2_ESM.docx]

| Additional file 2**:** Binomial Analysis of Factors Associated with Dental Caries (yes/no) (GEE) | | | | | |
| --- | --- | --- | --- | --- | --- |
| **Predictor** | **Category** | **Reference**  **Category** | **OR** | **95% CI**  **(Lower, Upper)** | **p-value** |
| Alignment | Not Aligned | Aligned | (1.01) | (0.60, 1.69) | .9600 |
| Child Grade | Grade 1 | Grade 3 | (1.64) | (1.10, 2.46) | 0.015* |
|  | Grade 2 |  | (1.11) | (0.76, 1.62) | 0.584 |
| Parent Gender | Female | Male | (0.28) | (0.06, 1.19) | 0.087 |
| Parent age | Continuous variable | - | (1.00) | (0.96,1.03) | 0.992 |
| Relationship with child | Guardian | Mother | (0.27) | (0.03, 2.39) | 0.240 |
|  | Grandparent |  | (0.25) | (0.01, 3.57) | .3120 |
|  | Father |  | (0.23) | (0.05, 1.02) | 0.054 |
| Education level | Postgraduate | ≤Secondary school | (0.73) | (0.39, 1.37) | 0.330 |
|  | Bachelor |  | (1.05) | (0.75, 1.46) | 0.774 |
| 1. Employment status | 1. Full-time employment | Unemployed / Homemaker / Retired | (0.65) | (0.43, 0.96) | 0.032* |
|  | 1. Self-employed |  | (0.69) | (0.16, 2.99) | 0.627 |
|  | 1. Part-time employment |  | (1.22) | (0.69, 2.16) | 0.475 |
|  | 1. Student |  | (0.47) | (0.08, 2.59) | 0.392 |
| Adults currently live in your household | One adult |  | (0.75) | (0.34, 1.62) | 0.469 |
|  | Two adults | More than four adults | (1.30) | (0.65, 2.61) | 0.455 |
|  | Three adults |  | (0.73) | (0.30, 1.76) | 0.489 |
|  | Four adults |  | (0.77) | (0.33, 1.81) | 0.562 |
| Number of children <16 years | Continuous variable | - | (0.76) | (0.65, 0.89) | 0.001* |
| Total Family Income | Less than 2500 | Above 15,000 | (0.12) | (0.04, 0.37) | <.001* |
|  | 2500–5000 |  | (0.35) | (0.23, 0.54) | <.001* |
|  | 5000–10000 |  | (0.25) | (0.14, 0.43) | <.001* |
|  | 10000–15000 |  | (0.41) | (0.23, 0.70) | 0.001* |
| How often does your child brush their teeth |  | Less than once a day |  |  |  |
|  | Several times a day |  | (0.81) | (0.54, 1.21) | 0.315 |
|  | Once a day |  | (0.98) | (0.61, 1.59) | 0.956 |
| Biscuits & Cakes consumption | At least once a day | Once a month or never | (0.31) | (0.07, 1.38) | 0.127 |
|  | At least once a week |  | (0.21) | (0.04, 1.00) | 0.050* |
| Fresh fruit  consumption | At least once a day | Once a month or never | (0.79) | (0.27, 2.25) | 0.664 |
|  | At least once a week |  | (0.66) | (0.28, 1.57) | 0.355 |
| Jam/honey  consumption | At least once a day | Once a month or never | (1.03) | (0.57, 1.86) | 0.910 |
|  | At least once a week |  | (1.21) | (0.95, 1.53) | 0.110 |
| Sweets/candy  consumption | At least once a day | Once a month or never | (1.07) | (0.48, 2.39) | 0.855 |
|  | At least once a week |  | (1.21) | (0.55, 2.67) | 0.621 |
| Chewing gum containing sugar  consumption | At least once a day | Once a month or never | (0.50) | (0.23, 1.09) | 0.082 |
|  | At least once a week |  | (1.05) | (0.75, 1.46) | 0.753 |
| Lemonade, Coca Cola or other soft  drinks consumption | At least once a day | Once a month or never | (0.97) | (0.43, 2.18) | 0.943 |
|  | At least once a week |  | (1.03) | (0.57, 1.85) | 0.911 |
| Sugary drinks consumption | At least once a day | Once a month or never | (0.96) | (0.61, 1.50) | 0.872 |
|  | At least once a week |  | (1.09) | (0.70, 1.70) | 0.674 |

**^*p<0.05^**
