# Supplementary material for: The relationship of dental caries and weight status with adherence to school nutrition policies among public primary school children in Riyadh: a cross-sectional study
Source: BMC Public Health. 2025 Nov 10;25:3871. doi: 10.1186/s12889-025-24979-0 (PMC12604184; doi:10.1186/s12889-025-24979-0)
Supplement: Supplementary file 3 — Supplementary Material 3. [file 12889_2025_24979_MOESM3_ESM.docx]

| Additional file 3: Binomial Analysis of Factors Associated with overweight and obesity (yes/no) (GEE) | | | | | |
| --- | --- | --- | --- | --- | --- |
| **Predictor** | **Category** | **Reference**  **Category** | **OR** | **95% CI**  **(Lower, Upper)** | **p-value** |
| Alignment | Not Aligned | Aligned | 0.80)) | (0.57, 1.13) | .2070 |
| Child Grade | Grade 1 | Grade 3 | 2.08)) | (0.96, 4.49) | 0.060 |
|  | Grade 2 |  | (1.01) | (0.72, 1.43) | 0.929 |
| Parent Gender | Female | Male | (0.84) | (0.59, 1.20) | 0.363 |
| Parent age | Continuous variable | - | (0.99) | (0.97, 1.02) | 0.929 |
| Education level | Postgraduate | ≤Secondary school | (1.03) | (0.47, 2.25) | 0.933 |
|  | Bachelor |  | (1.08) | (0.73, 1.59) | 0.691 |
| 1. Employment status | 1. Full-time employment | Unemployed / Homemaker / Retired | (1.34) | (0.89, 2.01) | 0.152 |
|  | 1. Self-employed |  | (2.30) | (0.61, 8.72) | 0.217 |
|  | 1. Part-time employment |  | (4.95) | (1.11, 21.88) | 0.035* |
|  | 1. Student |  | (0.93) | (0.29, 2.90) | 0.904 |
| Adults currently live in your household | One adult |  | (1.45) | (0.70, 2.99) | 0.308 |
|  | Two adults | More than four adults | (1.05) | (0.72, 1.54) | 0.777 |
|  | Three adults |  | (0.77) | (0.46, 1.30) | 0.333 |
|  | Four adults |  | (1.31) | (0.74, 2.33) | 0.349 |
| Number of children <16 years | Continuous variable | - | (0.85) | (0.74, 0.99) | 0.042* |
| Total Family Income | Less than 2500 | Above 15,000 | (1.03) | (0.46, 2.29) | 0.939 |
|  | 2500–5000 |  | (1.62) | (0.91, 2.88) | 0.094 |
|  | 5000–10000 |  | (1.57) | (1.08, 2.28) | 0.017* |
|  | 10000–15000 |  | (1.27) | (0.84, 1.91) | 0.244 |
| Biscuits & Cakes consumption | At least once a day | Once a month or never | (2.07) | (1.10, 3.88) | .022*0 |
|  | At least once a week |  | (3.05) | (1.49, 6.22) | 0.002* |
| Fresh fruit  consumption | At least once a day | Once a month or never | (0.68) | (0.14, 3.20) | .6340 |
|  | At least once a week |  | (0.61) | (0.14, 2.60) | 0.511 |
| Jam/honey  consumption | At least once a day | Once a month or never | (0.60) | (0.32, 1.12) | 0.114 |
|  | At least once a week |  | (0.62) | (0.47, 0.83) | 0.001* |
| Sweets/candy  consumption | At least once a day | Once a month or never | 0.94)) | (0.34, 2.55) | .9060 |
|  | At least once a week |  | (0.81) | (0.37, 1.79) | .6110 |
| Chewing gum containing sugar  consumption | At least once a day | Once a month or never | (1.74) | (0.78, 3.84) | .1690 |
|  | At least once a week |  | (1.24) | (0.83, 1.86) | .2790 |
| Lemonade, Coca Cola or other soft  drinks consumption | At least once a day | Once a month or never | (0.47) | (0.29, 0.77) | 0.003* |
|  | At least once a week |  | (0.86) | (0.59, 1.27) | .4690 |
| Sugary drinks consumption | At least once a day | Once a month or never | (0.63) | (0.40, 0.97) | .037*0 |
|  | At least once a week |  | (0.63) | (0.40, 0.98) | 0.042* |

**^*p<0.05^**
